# Feasibility Study on the Use of Infrared Thermography to Classify Fattening Pigs into Feeding Groups According Their Body Composition

**DOI:** 10.3390/s20185221

**Published:** 2020-09-13

**Authors:** Alexandra Lengling, Antonius Alfert, Bernd Reckels, Julia Steinhoff-Wagner, Wolfgang Büscher

**Affiliations:** 1 Institute of Agricultural Engineering, University of Bonn, 53115 Bonn, Germany; antonius.alfert@web.de (A.A.); buescher@uni-bonn.de (W.B.); 2 Institute for Animal Nutrition, University of Veterinary Medicine Hanover, Foundation, 30173 Hanover, Germany; Bernd.Reckels@tiho-hannover.de; 3 Institute of Animal Science, Process-und Product Management in Animal Production Group, University of Bonn, 53115 Bonn, Germany; jste@itw.uni-bonn.de

**Keywords:** body surface temperature, performance groups, pig husbandry, sorting gate, infrared images, thermal isolation, resource-efficient feeding

## Abstract

Fattening pig husbandry and associated negative environmental impacts due to nitrogen inputs by ammonia emissions are current issues of social discussion. New resource-efficient feeding systems offer great potential to reduce excess nutrient inputs into the environment. Using ultrasound measurements, fattening pigs can be divided into performance groups based on their backfat/muscle ratio to feed them according to their nutritional needs. Ultrasound measurements are not suitable for practical use, so alternatives have to be found. As a non-invasive, contactless method, infrared thermography offers many advantages. This study investigated whether infrared thermography can be used to differentiate between “fat” and “lean” animals. Two evaluation methods with different measurement spot sizes were compared. During a fattening period, 980 pigs were examined three times with an infrared camera. Both methods showed significant differences. Body surface temperature was influenced by factors like measurement spot size and soiling of the animals. Body surface temperature decreased (−5.5 °C), while backfat thickness increased (+0.7 cm) in the course of the fattening period. Significant correlations (*R* > |0.5|; *p* < 0.001) between both parameters were found. Differentiation between “fat” and “lean” animals, based on temperature data, was not possible. Nevertheless, the application of thermography should be investigated further with the aim of resource-efficient feeding. The results of this feasibility study can serve as a basis for this.

## 1. Introduction

In Germany, 95% of ammonia emissions originate from agriculture. Pig husbandry takes the second largest proportion [1]. The increasing spatial concentration of pig farms [2], and increasingly restrictive regulations regarding nitrogen inputs into the environment [3], make it necessary to find new possibilities to reduce nitrogen input and ammonia emissions. New feeding strategies offer great potential, especially in pig husbandry. Since only about 30% of ingested protein is utilized efficiently by the animal, and most of the nitrogen is excreted, it is urgently necessary to feed the animals according to their nutritional needs [4]. The prevention of luxury consumption of animals with a high feed intake capacity is desirable from an environmental and economic aspect. With conventional phase feeding systems, the diets are based on the average requirements of the whole animal group. Animals with feed intake capacity and nutritional needs above or below average are consequently not fed efficiently [5]. In their study, Reckels et al. [6] showed that fattening pigs can be divided into performance groups according to their individual body compositions. Those were determined by the ratio of backfat thickness and diameter of *Musculus longissimus dorsi* using ultrasound examinations. Furthermore, Lengling et al. [7] were able to demonstrate that feed intake of fattening pigs can be controlled by the crude fiber content of the diet, as both parameters correlate negatively [7]. In terms of resource-efficiency and based on the results of Reckels et al. [6] and Lengling et al. [7], feeding according to individual performance groups is desirable. However, ultrasound examinations are not suitable for practical use. New technologies have to be developed, which automates the classification of the animals into performance groups.

Sorting gates have already been used for several years to differentiate animals according to their bodyweight [8]. They can be used in large group housing systems of gestating sows [9] and fattening pigs, respectively. By the sorting gate, the lying area is separated from two different feeding areas. If the animals want to enter the feeding area, they have to pass the sorting gate. Using optical and mechanical weight determination, the animals’ bodyweight is recorded several times a day. According to those data, the animal is given access to feeding area A, or feeding area B, where different diets can be offered [5].

Infrared thermography (IRT) enables the measurement of heat radiation from objects or organisms. The IRT is being used increasingly in animal husbandry and veterinary medicine. As a non-invasive method, which can be repeated as often as required, it offers many advantages [10]. In reproductive medicine, IRT is used in sows to make conclusions about diseases like the mastitis-metritis-agalactia complex [11]. Changes in metabolism, that are due to a change in feed intake, or feed composition can be detected with IRT, respectively [12]. Several studies investigated the possibility of early detection of febrile animals, due to a change in body surface temperature [13,14].

The different thermal conductivity of fat and muscle tissue has already been investigated in many studies [15,16]. Henriques and Moritz reported thermal conductivity values of 11 × 10^−4^ (cal cm^−1^ s^−1^ °C^−1^) for porcine muscle tissue, while for porcine fat tissue, they reported 3.8 × 10^−4^ (cal cm^−1^ s^−1^ °C^−1^) [17]. Similar results have been presented by Breuer [18]. Fat tissue shows significantly lower thermal conductivity than muscle tissue, which is related to the different water content [19].

In the present study, it was examined, for the first time, whether infrared thermography is suitable to divide fattening pigs into performance groups. Firstly, it was hypothesized that different backfat thicknesses lead to differences in body surface temperature of the animals, as fat tissue acts as a thermal isolator [20]. Those differences should be visualized by infrared images and enable them to distinguish between fat and lean animals. Secondly, it was assumed that the infrared images would lead to a comparable grouping as obtained with the ultrasound examinations described by Reckels et al. [6] and Lengling et al. [7]. Furthermore, two different evaluation methods for the infrared images were compared to determine which method could be more suitable. This feasibility study should contribute to a new resource-efficient feeding concept. A technical extension of established sorting gate systems with an infrared camera could enable new options in feeding and animal health management.

## 2. Materials and Methods

### 2.1. Animals and Housing

The study was carried out from August to December 2019 on a pig fattening farm in Lower Saxony, Germany. For the trial, 980 fattening pigs with an average bodyweight of 25.8 kg were housed in. The males were castrated as suckling pigs, and all animals were cross-breed products of the Genesus F1 sow (Yorkshire x Landrace) and a Canadian Duroc boar. Each pig was equipped with a Radio-Frequency-Identification (RFID) ear tag on day three after housing, which enabled individual identification.

**Figure 1:**
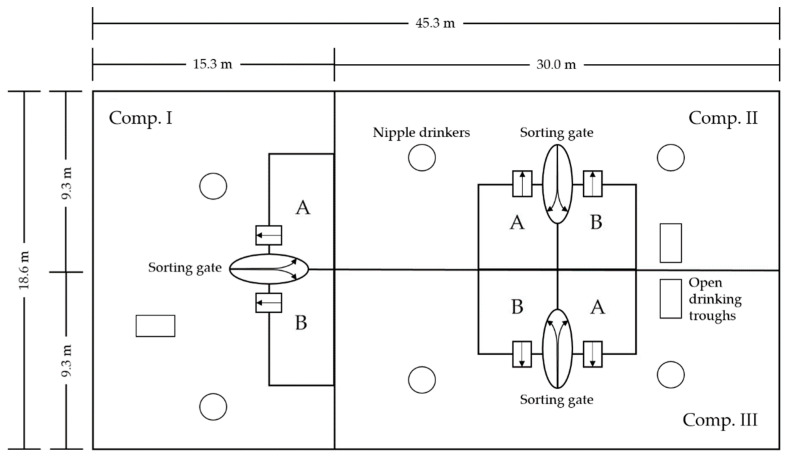
Outline of the experimental barn with its three compartments (Comp. I–III). Each compartment was equipped with two feeding areas (**A** and **B**, respectively), which could be reached by the animals of each compartment via a sorting gate and left via an exit door.

The barn was designed for large group housing and subdivided into three compartments with a total surface area of 841 m^2^. Since the prescribed area per animal has to be at least 0.75 m^2^ [21], the barn had a total capacity of 1121 animals. Due to the fact that only 980 pigs were housed in, there was a usable surface area of 0.85 m^2^ per animal. Activity and lying areas were spatially separated from two feeding areas (feeding area “A” and “B”) per compartment. A sorting gate of the company Hoelscher + Leuschner (^®^Hoelscher + Leuschner GmbH and Co. KG, Emsbueren, Germany) in each compartment, connected the different areas. The barn was force ventilated. Fresh air was continuously supplied along the eaves, while exhaust air was decentralized extracted over the floor by means of three exhaust fans with a diameter of 1090 mm. Approximately 60% of the barn were equipped with a concrete slatted floor with a void percentage of 15%. The remaining 40% were equipped with a structured plastic slatted floor (Comfi-Floor, Hoelscher + Leuschner GmbH and Co. KG, Emsbueren, Germany) with a reduced void percentage of 3.8%. Figure 1 gives an outline of the barn.

### 2.2. Feeding

#### 2.2.1. Feeding Groups

The animals were divided into three groups with two feeding groups, each according to their body composition. The grouping was done with an average bodyweight of the animals of 50 kg. Bodyweight was determined by all animals using a weighbridge inside the sorting gate. Additionally, the ratio between backfat thickness and the diameter of *Musculus longissimus dorsi* was measured by using ultrasound examinations, as described by Reckels et al. [6] and Lengling et al. [7]. Group 1 in compartment I contained 330 randomly selected animals and represented an average group. The remaining 650 animals were firstly divided into group 2 (compartment II) and group 3 (compartment III), according to their bodyweight. Group 2 contained approximately the 50% heaviest (*n* = 328) and group 3 approximately the 50% lightest (*n* = 322) animals. Each group was further divided into two feeding groups. In group 1, the feeding groups consisted of the approximately 50% heaviest and 50% lightest animals. Since groups 2 and 3 have already been separated according to the bodyweight, those groups were subdivided related to the ratio between backfat thickness and diameter of *Musculus longissimus dorsi* in “fat” (≥0.19) and “lean” (<0.19). Thus, for group 2, the subgroups were “heavy lean (HL)” and “heavy fat (HF)”; and for group 3, the subgroups were “light lean (LL)” and “light fat (LF)” [6,7]. Table 1 gives an overview of the feeding groups.

#### 2.2.2. Feeding Technology

Due to the two spatially separated feeding areas in each compartment, it was possible to feed each feeding group with an individual diet. The animals had to pass the sorting gate to change from lying area to the feeding areas. The sorting gate consists of an entrance door and two exit doors, each leading to one of the two feeding areas. By means of the RFID ear tag recognition, each animal could be identified when entering the gate. Using a weighbridge and three-dimensional (3D) camera-technology, the animal’s individual weight and body condition data were recorded mechanical and optical every time it passed the sorting gate. Optical weight is calculated by measuring the height, width, and length of the animal with the assistance of special software (optiSORT, Hoelscher + Leuschner GmbH and Co. KG, Emsbueren, Germany). Cielejewski et al. [8] verified the correlation between optical and mechanical weight determination. Depending on the data, the animals were directed to either feeding area “A”, or feeding area “B”. The animals were fed with a liquid feeding system of the company Hoelscher + Leuschner (^®^Hoelscher + Leuschner GmbH and Co. KG, Emsbueren, Germany) *ad libitum* for the entire fattening period. The liquid feed for the fattening pigs was composed of different components. Corn-Cob-Mix (CCM) and Triticale whole-plant-silage (WPS) were used as the farm’s own components. Two different supplementary feed and soybean oil were purchased from a German feed company. Detailed chemical composition of the feeding components is given in Table S1 in the Supplementary Materials (see Table S1: Chemical composition of supplementary feed “SF1”, and “SF2”, as well as “Soybean Oil”, according to the declaration (88% dry matter content; DM). Triticale whole-plant-silage (WPS) and the Corn-Cob-Mix (CCM) were analyzed in the Institute for Animal Nutrition Hanover (88% DM)). The diets of the feeding groups only differed in their crude fiber contents. Thus, the “fat” animals were given a higher crude fiber content in order to limit feed intake and avoid luxury consumption. The two animal groups in compartment I received the same diets as the “lean” animal groups in compartment II and III. As a crude fiber component, the WPS was used. All diets had an equal amount of energy and nitrogen. Water was provided by open drinking troughs and nipple drinkers, additionally to the water provided with the liquid feeding.

### 2.3. Infrared Thermography

Thermographic measurements were carried out on three dates during the fattening period (fattening day 32, 61, and 109) with 1828 examined animals in total. At day 32 and 109, all animals in the barn were examined (average bodyweight 53.1 ± 9.0 kg and 109.6 ± 8.8 kg). Because the first amount had already been marketed for slaughtering, there were fewer animals on day 109. At day 61 (average bodyweight 72.5 ± 8.6 kg), only the animals from group 1 were investigated, which is related to the parallel ultrasound examinations to which the thermographic measurements were adjusted. In addition to the thermographic measurements for each animal, the ear tag number, a reference temperature, and parameters of body composition were recorded.

The infrared thermography was performed with a VarioCAM (InfraTec GmbH, Dresden, Germany). The camera covered a spectral range from 7.5 to 14 µm and a temperature range from −40 to 1200 °C. The measurement accuracy was ±2%. The infrared images of the animals were taken in the sorting gate of compartment I. The animals were successively moved into the sorting gate for the measurements. It was tried to handle the animals as calm as possible to avoid an increased stress level. Since the animals were used to enter and remain in the sorting gate, this was not a new situation for them. No fixation techniques to the animals were necessary inside the sorting gate. In order to ensure that all images are taken in the same distance and angle, the infrared camera was fixed to the sorting gate using a bracket. The camera was, thus, located at a total height of 1.81 m above the floor of the gate. The emission level was set at a constant of 0.98, as described for pigskin by Gerß [14]. The measuring angle was approximately vertical to the measuring object. Due to the permanently mounted technology of the sorting gate, a completely vertical angle was not possible. The camera was operated manually for each image, and the images were saved automatically. By the individual ear tags, the images could be exactly assigned to the animals. In total, 529 animals could be identified, which were investigated on days 32 and 109, while 156 animals were assigned to the measurements on all three measurement days. Ultrasound measurements, as described by Reckels et al. [6] and Lengling et al. [7], were performed on the animals at the same time. Feeding and ultrasound data will be reported in detail elsewhere and were only used in times of a high variance to evaluate the infrared thermography for classification.

#### 2.3.1. Evaluation of Thermograms

**Figure 2:**
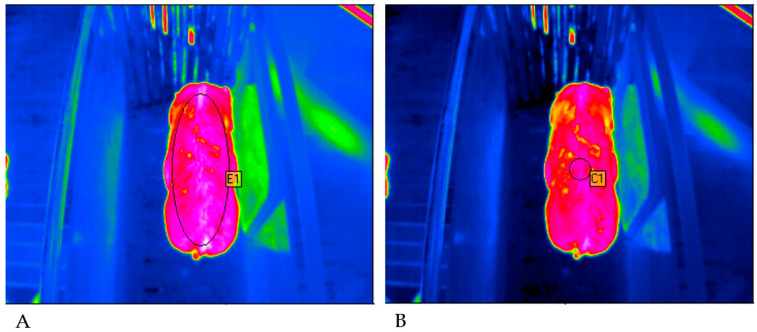
Comparison of the two evaluation methods. (**A**) First method (ME, maximal ellipse) with an elliptical measurement spot placed over the maximum possible area of the pig’s body. (**B**) Second method (SC, standardized circle) with a standard circular measurement spot at the height of the last rib of the animal (©Alfert).

Two methods were used for the evaluation of the thermograms. A comparison of those was made in order to determine, which method is more suitable for the research question. Figure 2 shows the evaluation methods used on the basis of a thermogram.

The evaluation of the thermograms was done with IRBIS^®^ Software (InfraTec GmbH, Dresden, Germany). The evaluation methods differed in the size of the measuring spot. For the first method, the spot was adapted to the size of each animal individually. Therefore, it had an elliptical geometry in order to cover the maximal possible area of the animal’s body (method 1 = maximal ellipse = ME). For the second method, the measurement spot was standardized (method 2 = standardized circle = SC). A circle with a radius of 3.5 cm and a base area of 38.5 cm^2^ was used. The measurement spot was placed in the same position as the ultrasound measurements at the height of the last rib.

**Figure 3:**
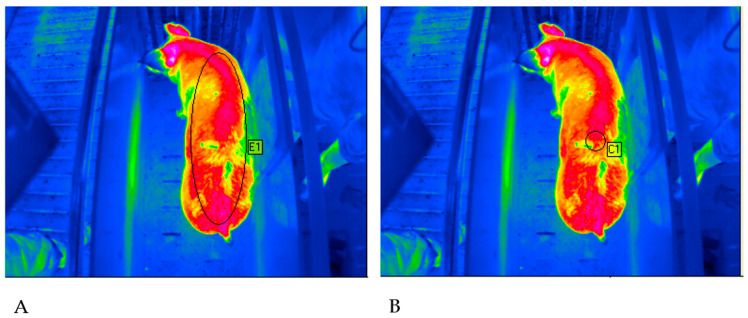
Thermogram of a fattening pig with visible soiling within the measurement spots. (**A**) Evaluation method ME (maximal ellipse). (**B**) Evaluation method SC (standardized circle) (©Alfert).

Animals were excluded from data analysis, due to an increased degree of soiled skin. Whenever contamination was detected within the measuring spot, an animal was classified as soiled (Figure 3).

Consequently, animals which were classified as soiled in SC, automatically were classified as soiled in ME, respectively. Table 2 summarizes the number of investigated and excluded animals on the three measurement days.

#### 2.3.2. Reference Temperature and Climatic Conditions

In order to take a reference value of the temperature of each animal, an infrared thermometer IR-1001A (Voltcraft, Hirschau, Germany) was used. Reference temperature was taken to obtain a comparative value to the temperature data received with the thermograms. It also represents the body surface temperature and was not used to represent the body core temperature of the animals. The measurement accuracy of the thermometer was ±1.5% for a temperature range of −20 to 200 °C. The reference temperature was measured on the skin surface of the pigs, at the height of the last rib, respectively. The emission level was also 0.98. The distance to the measuring point was 30 cm. With a ratio of measuring distance and measuring spot size of 50:1, the measuring spot size was 0.6 cm^2^. Due to the limited space in the sorting gate and the intention to keep the animals as short and unstressed as possible for the measurements, a rectal or orbital temperature measurement as a reference value was not feasible.

Climatic parameters, like temperature and relative humidity, can influence IRT measurements [22,23]. For this reason, these data were recorded during the fattening period with data loggers Testo 174 H (Testo SE and Co. KGaA, Lenzkirch, Germany) continuously every five minutes.

### 2.4. Statistical Analysis

The infrared images were evaluated descriptively by minimum, mean, and maximum with the IRBIS^®^ Software (InfraTec GmbH, Dresden, Germany). All charts were created with Microsoft (MS) Office Excel (Microsoft^®^ Office Professional Plus 2013). Statistical analysis was done with SAS 9.4 (SAS, 2016). Results are presented as mean values ± standard error (SE). Body composition and reference temperature data of the animals, which were identified on all three measurement days, were analyzed by a General Linear Model with measurement days as a fixed effect. Body surface temperature data were analyzed by the Mixed Model with soiling status, evaluation method, and measurement day, as well as their interactions as fixed effects and individual pig as a random effect. The post-hoc Tukey multiple comparison tests were performed to determine statistically significant differences. Correlations were performed according to Pearson with *R* > |0.3| as weak, *R* > |0.5| as mean, and *R* > |0.8| as strong correlation. All statements of statistical significance were based on *p* ≤ 0.05.

## 3. Results

### 3.1. Comparison of Evaluation Methods

For comparison of the two evaluation methods, measured temperature minima, mean, and maxima, were evaluated. For all temperature measurements, significant differences were found between method ME and SC. In general, body surface temperature determined with ME was lower than with SC. The highest difference was found for the temperature minimum. For ME, the mean of temperature minima for all three measurement days was 28.06 °C ± 0.13, whereas for SC, it was 31.91 °C ± 0.07. The mean of mean temperature values for all measurement days were 33.07 °C ± 0.06 and 33.44 °C ± 0.07 (ME and SC), respectively, while the mean of maxima were 34.92 °C ± 0.05 and 34.32 °C ± 0.05 (ME and SC). Method ME consequently showed a temperature range between mean minimum and maximum temperature of 6.86 °C, while SC had a temperature range of 2.41 °C. A significant difference between ME and SC could be found for all three measurement dates, respectively.

In order to investigate the influence of contamination on the temperature data, body surface temperature of soiled and non-soiled animals were compared. In general, the body surface temperature of the soiled animals was lower than of the non-soiled animals. These results were found for ME and SC, respectively. For example, for ME, the mean temperature of the soiled animals was 32.81 °C, while the non-soiled animals showed a mean temperature of 34.19 °C (on measurement day 1; *p* < 0.01). For method SC, similar significant differences between the soiled and non-soiled animals were found. Temperature decreased from measurement day 1 to 3, independent of soiling status or evaluation method. Table 3 shows the results in detail.

**Figure 4:**
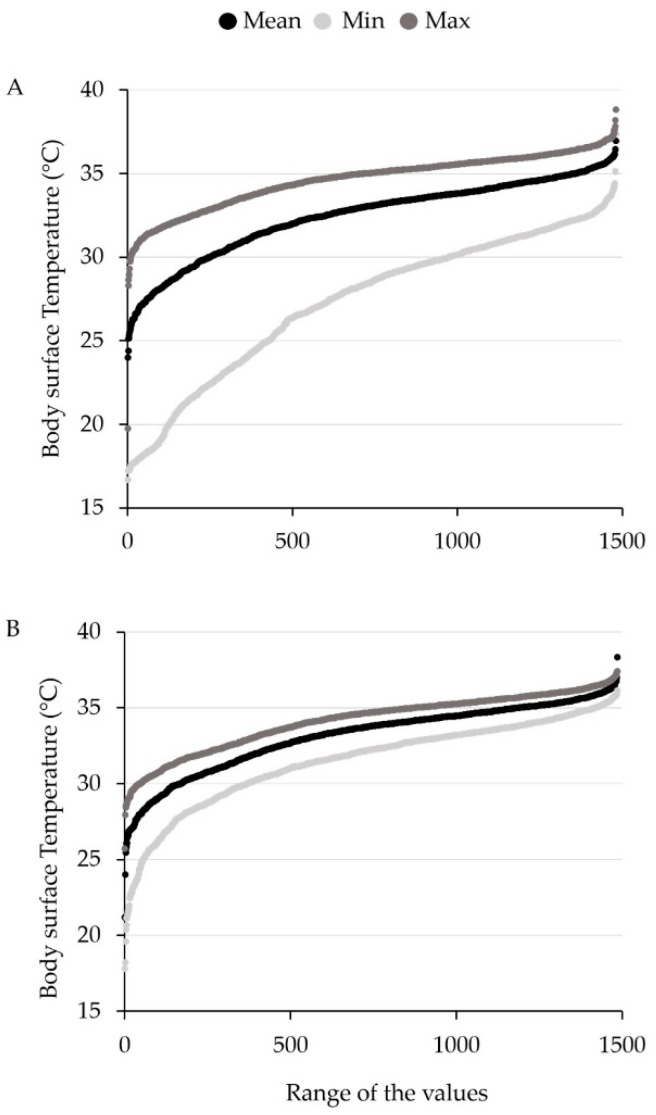
Cumulative distribution of all measured values for minimum, mean, and maximum body surface temperature (°C) for evaluation method ME (**A**) and SC (**B**).

For both methods, the temperature maximum showed the lowest variance. In contrast, the temperature minimum showed a higher variance, especially for ME, and was more influenced by animal soiling than mean and maximum temperature values. Figure 4 shows the cumulative distribution of all measured temperature values for minimum, mean, and maximum for method ME and SC, respectively.

The two evaluation methods showed significant differences (*p* < 0.05) with regard to evaluable thermograms of the animals. For ME, 30.2% of the thermograms were classified as soiled, while for SC, only 12.6% were excluded, due to contamination. Thus, less than half as many thermograms were excluded, due to contamination in SC compared to ME.

For both evaluation methods, strong, significant correlations with the reference temperature could be observed for all temperature parameters with *r* ≥ 0.8 and *p* ≤ 0.001.

Due to a technical defect, no climate data were available for measurement 1. During measurement 2, a higher relative humidity was measured in compartment I. Further significant differences could not be determined for indoor temperature and relative humidity between the compartments on measurements 2 and 3 (*p* > 0.05).

### 3.2. Body Composition and Body Surface Temperature

Table 4 summarizes the results of body composition and reference temperature measurements of the animals, which were identified on all three measurement days (*n* = 156). As those animals, all belong to group 1, and this was an average group of all animals in the barn, this data can be seen as representative for all animals.

During the fattening period, the body composition of the animals changed, and the effects of those on the temperature data were observed. From measurements 1 to 3, a gain in bodyweight of 53.35 kg per pig and a mean reduction of reference temperature of 5.57 °C was observed. Thus, the reference temperature decreased from 36.72 °C (measurement 1) to 31.15 °C (measurement 3). Backfat/muscle ratio increased during fattening period from 0.19 ± 0.03 to 0.27 ± 0.05.

**Figure 5:**
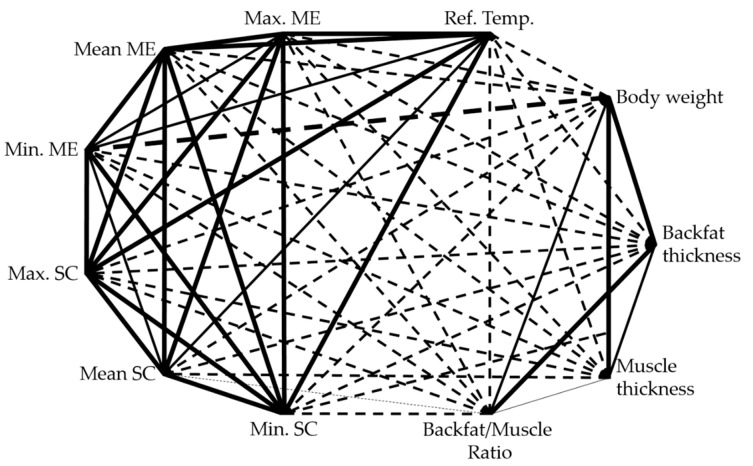
A significant correlation (*p* < 0.0001) for all measurements on all three measurement days between body surface temperature measured with ME and SC, reference temperature, and body condition parameters like bodyweight, backfat thickness, muscle thickness, and backfat/muscle ratio. Continuous lines indicate positive correlations, and dotted lines indicate negative correlations. The thicker the connecting line, the stronger the correlation, with *R* > |0.3| as weak, *R* > |0.5| as mean and *R* > |0.8| as strong correlation.

Data analysis showed significant correlations between all measurement parameters (temperature values, as well as body condition parameters) for all measurements on all three measurement days (Figure 5.)

**Figure 6:**
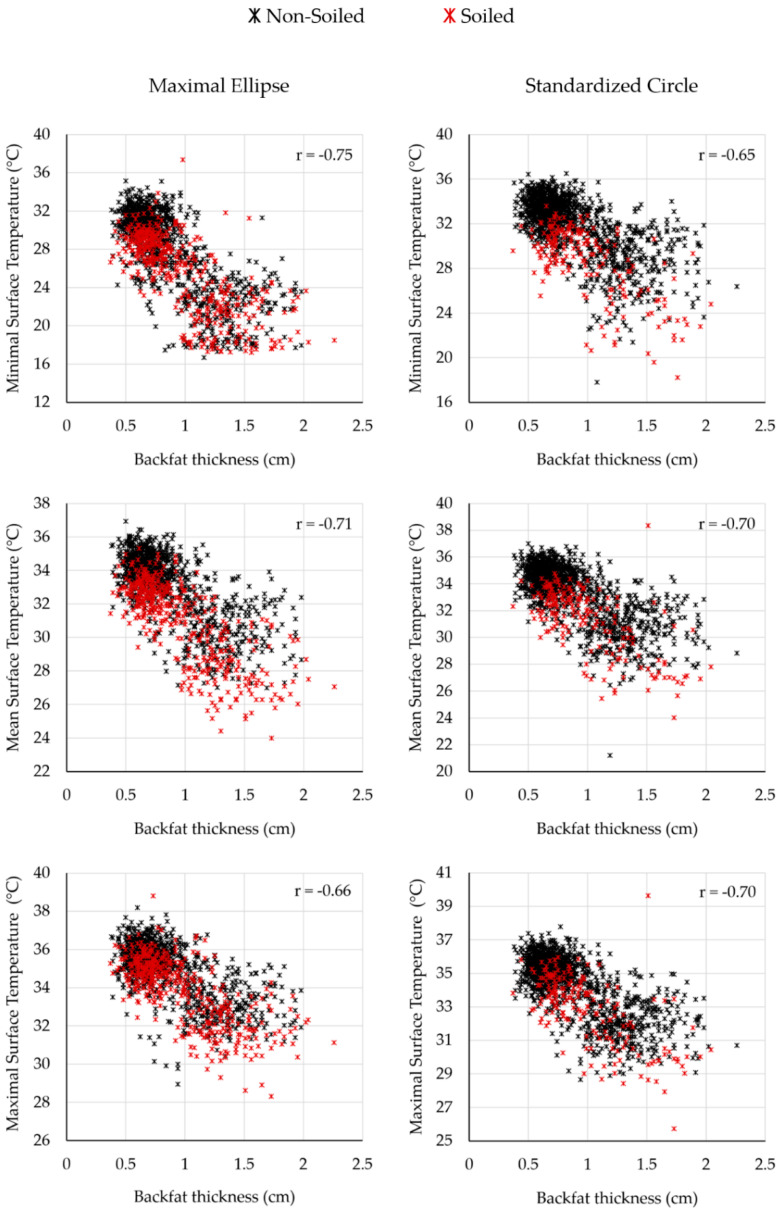
Correlation between body surface temperature (°C) and backfat thickness (cm) for soiled and non-soiled animals with *n* = 1457 in total. Left column: evaluation method ME; right column: evaluation method SC (*p* < 0.001).

One of the research questions was whether there is a visible correlation between the backfat thickness and the body surface temperature. Reference temperature and body surface temperature determined by the thermograms decreased with increasing body condition values, respectively. Figure 6 shows the correlation between body surface temperature and backfat thickness, according to the two evaluation methods, and differentiated between the soiled and non-soiled animals for all measurement days. All figures show a negative trend. Similar correlation values were found for both methods and all temperature parameters.

### 3.3. Comparison of Body Surface Temperature and Feeding Group

Body condition values and body surface temperatures determined by thermography were compared for the different compartments and each feeding group. On measurement 1, no significant differences were found for body surface temperature values, neither between the compartments nor between the feeding groups within the compartments (*p* > 0.05). Nevertheless, a significant interaction for temperature maximum determined with ME could be found (*p* = 0.05). Except for bodyweight (heavy animals 67.8 kg; light animals 51.6 kg; *p* < 0.05), no significant differences were found between the heavy and the light animals in compartment I. Body condition values differed significantly between compartment II and III (*p* < 0.05). The backfat thickness and backfat/muscle ratio also differed significantly between the feeding groups within the same compartment. A noteworthy interaction was determined for backfat thickness, respectively. Table 5 shows the mean values and standard error for compartment II and III of measurement 1, according to the feeding groups.

During measurement 2, only the animals in compartment I were investigated. No significant difference between the two feeding groups could be observed either (*p* > 0.05). On average, the body surface temperature of the light animals was 32.81 °C ± 0.30 and 33.64 °C ± 0.30 (method ME and SC). For the heavy animals mean temperature was 33.10 °C ± 0.23 and 33.61 °C ± 0.22 (method ME and SC).

On measurement 3, the body surface temperature of the animals in compartment I differed significantly from those in compartment II and III (*p* < 0.05). On average, the body surface temperature was lower in the compartment I compared with the other two compartments. Except for bodyweight (heavy animals 113.3 kg; light animals 106.4 kg; *p* < 0.05), no significant differences were found between the heavy and the light animals in compartment I. Comparing compartment II and III, significant differences were found between the feeding groups for backfat, muscle, and backfat/muscle ratio (*p* < 0.05). Bodyweight differed significantly between those two compartments, but not between the feeding groups within the compartments. For body surface temperature, no significant differences were found, neither between the compartments II and III, nor between the feeding groups within the compartments (*p* > 0.05). However, as shown for measurement 1, a significant interaction for temperature maximum in ME could be determined for measurement 3, respectively (*p* = 0.05). Table 6 shows the mean values and standard error for compartment II and III of measurement 3, according to the feeding groups.

## 4. Discussion

As a non-invasive method, the infrared thermography offers many possibilities for temperature measurement, especially in livestock husbandry and veterinary medicine. Until now, IRT has not been used commonly in fattening pig husbandry. Technical and environmental parameters influencing the measured values must be identified and taken into account for practical use [24]. For an automated and standardized application of IRT in practice, a suitable and reliable evaluation method is essential. Few authors describe the different possibilities of evaluating thermal images and the advantages and disadvantages of those [25,26,27]. Therefore, two evaluation methods were compared in this study. The two methods differed significantly from each other for all measured parameters. In her study, Glas [26] also used different evaluation methods. Using a polygon measuring tool, the maximum area of the object is investigated. Therefore, the individual limits of each object are manually circumvented. Due to the large dataset used in this study, this was not practical. Instead, an elliptical shape was used, which described the pigs’ corpus almost completely.

For the second method, a standardized, circular measuring spot at the level of the last rib was used. In her study, Glas [26] also uses a standardized measuring spot as second method. In method SC, the difference between the minimum and maximum temperature was reduced compared to method ME. The percentage of animals classified as soiled was significantly lower in method SC, than in method ME, respectively.

In this study, the reference temperature was measured using an infrared thermometer. On average, the two methods did not differ significantly in their deviation to the reference temperature. Both methods showed a strong correlation for all temperature parameters with the reference temperature. Schmidt et al. [20] also showed a correlation between infrared thermometer and IRT. In general, the measured reference temperature was higher than the maximum temperature determined with IRT. Consequently, the temperature maximum showed the smallest deviation from the reference temperature compared to minimum and mean values. This indicates, that the body surface temperature of fattening pigs can be described most accurately by the temperature maximum of the thermal images. The largest difference between the two evaluation methods was shown for temperature minimum. Due to the physiological limits of heat tolerance (3–6 °C) and cold tolerance (15–25 °C) of pigs [28], the temperature maximum shows less variation compared to the temperature minimum. Factors like moisture, soiling, hair growth, or airflow can influence the temperature minimum, which makes it more sensitive to disturbances [22,23,25]. This fact also implies, that the temperature maximum is the most suitable parameter for measuring the body surface temperature. This can be confirmed by Traulsen et al. [11]. In their study, temperature maximum measured with IRT showed the best correlation to rectal temperature. Other studies show strong correlations between the rectal temperature and body surface temperature, respectively [13,29]. It should be considered, that rectal temperature measurement is an invasive and stressful process for the animals, which may lead to an increase, and thus, confounding of the body temperature [30]. With regard to animal welfare and economic aspects, stressful situations for the animals should be avoided, even though rectal measurement still constitutes the golden standard for measurement of the body core temperature. Considering the aim of an automated and continuous temperature measurement in the daily practice of fattening pig husbandry, contactless measurements with an infrared thermometer are preferable for this purpose.

As shown by Reckels et al. [6] and Lengling et al. [7], fattening pigs can be divided into performance groups according to their backfat/muscle ratio. In this study, it was investigated, for the first time, whether such a division based on body surface temperature is possible. As fat tissue acts as a thermal isolator [31], it was investigated, whether there is a correlation between backfat thickness and body surface temperature. By infrared thermography, a reduction of body surface temperature could be observed in the course of the fattening period. Backfat thickness increased over the same period, respectively. Significant correlations between both parameters were shown. Thus, the hypothesis could be verified. However, it should be noted that the body surface temperature can be influenced by external factors. Hair intensity and contamination of the skin (e.g., moisture, feed residues) reduce the surface temperature [23]. Both factors increase towards the end of the fattening period and could be related to temperature reduction. As shown, the body surface temperature of soiled animals was significantly lower compared to non-soiled animals. Since the body surface temperature decreases with increasing age of the animals, and at the same time, soiling increases, this fact must be taken into account. By using the temperature maximum for determining body surface temperature, the influence of soiling can be minimized. Direct air circulation can reduce body surface temperature [32]. Since the animals were examined inside the sorting gate, direct airflow can be almost completely excluded. The ambient temperature can also influence body surface temperature. This factor should be minimized by comparable internal temperatures between the compartments and the continuous control of it. Within the same compartment, it can be assumed that the ambient temperature has the same effect on all measurements, and consequently, a potential measurement error is balanced. During the study, ambient temperature was measured continuously, and no significant differences were found between the compartments. Nevertheless, cold ambient temperatures influence body surface temperature less than warm ambient temperatures. For this reason, the tested method is more suitable for cold ambient temperatures to visualize differences in body surface temperature, due to different fat layers. This factor must be taken into account when applying the method. In future studies, it has to be investigated how ambient temperature can be included in the evaluation or how its influence can be minimized.

The second hypothesis of this study was that classification into performance groups (based on infrared thermography) is possible, and would be comparable to the classification done with ultrasound examinations [6,7]. In the literature, no comparable investigations are described. No significant differences in body surface temperature between the groups could be found. Only group 1 in compartment I differed significantly from the other groups on measurement 3. However, even within the compartment, no differences could be detected between the two feeding groups, “light” and “heavy”. Since ultrasound measurements showed significant differences in the backfat/muscle ratio between “fat” and “lean” animals, the results suggest that differences in fat tissue do not affect thermal isolation, and thus, body surface temperature to the same extent. The differences in backfat- and muscle thickness between “fat” and “lean” animals lie within a range of a few millimeters [6]. It is possible that such small differences are not sufficient to produce a significant difference in surface temperature—even if they can be detected with ultrasound examinations. The reason why the temperatures in group 1 on measurement 3 were significantly lower compared to the other groups cannot be answered conclusively. The fact that there were no differences between the subgroups “heavy” and “light” within group 1 suggests, that external factors influenced the temperature evaluation. During the measurements, the conditions were tried to keep as standardized as possible to minimize disturbing factors. Nevertheless, there are some factors, like stress, which are difficult to determine and control. This could have influenced the results. Berry et al. [33] recommend a detailed recording of the present measurement conditions. However, inside a barn of pig husbandry, it might be difficult to control all disturbing factors.

## 5. Conclusions

Based on the results, method SC seems to be less susceptible to temperature variations and more resistant to factors, such as contamination. Additionally, the evaluation with a standardized measuring spot is less work-intensive, and thus, time-saving. However, it has to be clarified how representative a standardized measuring spot is in comparison to the whole measured object. Under this aspect, ME seems to be more suitable to represent the body surface temperature of the animal. Temperature maximum seems to be the most reliable parameter for the determination of body surface temperature, as it shows the lowest variance and the lowest difference to the measured reference temperature. Furthermore, the temperature maximum showed less influence by soiling than temperature mean and minimum. In conclusion, the combination of ME and temperature maximum can be considered the most reliable method. Nevertheless, the results should be confirmed by further studies.

In the present feasibility study, a correlation between body condition and surface temperature could be shown. However, a classification of different performance groups using infrared thermography was not possible. Nevertheless, as a non-invasive method, infrared thermography offers many advantages. A combination of the sorting gates with a thermal imaging camera would enable continuous data acquisition. Daily temperature profiles of the animals could be recorded, as the animals pass the sorting gate several times a day. Correlations between body development and body temperature could, thus, be visualized in more detail. The possibility of using infrared thermography for the described purpose cannot be excluded. With the aim of resource-efficient feeding, this technique should be investigated in further studies.

## Figures and Tables

**Table 1:** Classification of animals into feeding groups.

Compartment	Group	Feeding group	Number of Animals
I	1	Light	166
		Heavy	164
II	2	Heavy lean	164
		Heavy fat	164
III	3	Light lean	161
		Light fat	161

**Table 2:** The number of investigated and soiled animals (% of investigated animals) of the three measurement days (MD) (fattening day 32, 61, and 109) and in total for the entire period.

MD	Investigated Animals	Soiled Animals (%)		Non-Soiled Animals (%)
		SC	ME and SC *	
1	933	7.3	23.1	76.9
2	289	20.8	25.6	74.4
3	606	9.9	26.1	73.9
Total	1828	10.3	24.5	75.5

* ME = maximal ellipse; SC = standardized circle; Animals which were classified as soiled in SC consequently were classified as soiled in ME.

**Table 3:** Body surface temperature (mean ± SE (*n*)) of soiled and non-soiled fattening pigs determined by evaluation method ME (maximal ellipse) and SC (standardized circle) on three measurement days (MD).

MD	Body Surface Temperature (°C)	Evaluation Method			
		ME		SC	
		Soiled ^‡^	Non-Soiled	Soiled ^‡^	Non-Soiled
1	Min	28.87 ± 0.11 (215) ^A^	30.82 ± 0.07 (639) ^A,^*	30.12 ± 0.19 (68) ^A,#^	33.34 ± 0.04 (787) ^A,^ *^,#^
	Mean	32.81 ± 0.07 (215) ^A^	34.19 ± 0.04 (639) ^A,^*	32.67 ± 0.14 (68) ^A^	34.66 ± 0.06 (787) ^A,^ *^,#^
	Max	35.01 ± 0.06 (215) ^A^	35.65 ± 0.03 (639) ^A,^*	34.18 ± 0.13 (68) ^A,#^	35.33 ± 0.03 (787) ^A,^ *^,#^
2	Min	26.70 ± 0.18 (74) ^B^	26.88 ± 0.12 (129) ^B^	30.57 ± 0.16 (60) ^A,#^	32.16 ± 0.13 (145) ^B,^ *^,#^
	Mean	31.66 ± 0.09 (74) ^B^	32.95 ± 0.13 (129) ^B,^*	32.45 ± 0.13 (60) ^A^	33.63 ± 0.12 (145) ^B,^ *^,#^
	Max	34.74 ± 0.11 (74) ^A^	35.07 ± 0.14 (129) ^B^	33.60 ± 0.13 (60) ^B,#^	34.49 ± 0.12 (145) ^B,^ *^,#^
3	Min	21.05 ± 0.20 (158) ^C^	21.93 ± 0.16 (263) ^C,^*	24.76 ± 0.33 (60) ^B,#^	28.74 ± 0.13 (366) ^C,^ *^,#^
	Mean	28.23 ± 0.13 (158) ^C^	30.43 ± 0.09 (263) ^C,^*	28.44 ± 0.28 (60) ^B^	30.75 ± 0.09 (366) ^C,^ *
	Max	31.94 ± 0.12 (158) ^B^	33.10 ± 0.06 (263) ^C,^*	30.60 ± 0.24 (60) ^C,#^	32.10 ± 0.07 (366) ^C,^ *^,#^

^A, B, C^ different letters indicate differences (*p* < 0.01) among the measurement days 1–3 within the same soiling status and evaluation method. * stars indicate differences (*p* < 0.01) between the soiled and non-soiled groups within the same measurement day and evaluation method. ^#^ hash keys indicate differences (*p* < 0.01) between evaluation method ME and SC within the same soiling status and the same measurement day. ^‡^ soiling rating refers to the total visible top view of the pig independent of the evaluation method.

**Table 4:** Body composition and reference temperature data of the animals which were identified on all three measurement days (*n* = 156).

Parameter	MD	Mean	SE	Minimum	Maximum
Bodyweight (kg)	1	57.58 ^A^	1.334	39.4	70.4
	2	69.99 ^B^	0.752	42.6	89.7
	3	110.93 ^C^	0.841	91.4	160.6
Backfat (cm)	1	0.63 ^A^	0.012	0.43	0.97
	2	0.85 ^B^	0.017	0.37	1.56
	3	1.34 ^C^	0.022	0.83	1.98
Muscle (cm)	1	3.37 ^A^	0.044	2.65	4.52
	2	4.14 ^B^	0.04	3.36	5.52
	3	4.97 ^C^	0.04	3.95	6.15
Backfat/Muscle	1	0.19 ^A^	0.003	0.12	0.27
	2	0.21 ^B^	0.004	0.07	0.37
	3	0.27 ^C^	0.005	0.17	0.42
Reference Temp. (°C)	1	36.72 ^A^	0.121	34.1	38.8
	2	36.23 ^B^	0.162	32.5	39.8
	3	31.15 ^C^	0.171	25.5	36.5

^A, B, C^ different letters indicate significant differences (*p* < 0.05) between the measurement days within the same parameter. MD, measurement day; SE, standard error.

**Table 5:** Body condition values and body surface temperatures (mean ± SE (*n*)) for compartment II and III on measurement day 1, according to the feeding groups.

	Comp. II		Comp. III		*p*-Values (ANOVA)		
	Heavy/Lean	Heavy/Fat	Light/Lean	Light/Fat	Comp.	Feeding Group	Comp. × Feeding Group
Backfat (cm)	0.646 ± 0.007 (154) ^a^	0.817 ± 0.009 (150) ^a,^*	0.554 ± 0.007 (154) ^b^	0.691 ± 0.008 (154) ^b,^*	0.001	0.001	0.03
Muscle (cm)	3.83 ± 0.03 (154) ^a^	3.73 ± 0.03 (150) ^a^	3.42 ± 0.03 (154) ^b^	3.32 ± 0.03 (154) ^b^	0.001	0.002	n.s.
Backfat/Muscle	0.169 ± 0.001 (154) ^a^	0.219 ± 0.002 (150) ^a,^*	0.162 ± 0.001 (154) ^b^	0.208 ± 0.002 (154) ^b,^*	0.001	0.001	0.14
Bodyweight (kg)	56.76 ± 0.41(147) ^a^	57.15 ± 0.47 (142) ^a^	44.98 ± 0.28 (146) ^b^	45.91 ± 0.28 (149) ^b^	0.001	0.07	n.s.
Reference Temp. (°C)	36.99 ± 0.11 (146)	36.85 ± 0.10 (145)	36.97 ± 0.11 (145)	37.09 ± 0.10 (148)	n.s.	n.s.	n.s.
Min. Temp. ME (°C)	30.47 ± 0.16 (142)	30.46 ± 0.15 (139)	30.13 ± 0.19 (136)	30.37 ± 0.16 (142)	n.s.	n.s.	n.s.
Mean. Temp. ME (°C)	33.89 ± 0.10 (142)	33.75 ± 0.99 (139)	33.91 ± 0.09 (136)	33.97 ± 0.08 (142)	n.s.	n.s.	n.s.
Max. Temp. ME (°C)	35.53 ± 0.07 (142)	35.35 ± 0.07 (139)	35.46 ± 0.07 (136)	35.55 ± 0.07 (142)	n.s.	n.s.	0.05
Min. Temp. SC (°C)	33.08 ± 0.15 (143)	33.05 ± 0.13 (138)	33.11 ± 0.12 (136)	33.11 ± 0.12 (142)	n.s.	n.s.	n.s.
Mean Temp. SC (°C)	34.45 ± 0.09 (143)	34.30 ± 0.10 (138)	34.65 ± 0.24 (136)	34.54 ± 0.09 (142)	n.s.	n.s.	n.s.
Max. Temp. SC (°C)	35.25 ± 0.08 (143)	35.11 ± 0.08 (138)	35.23 ± 0.07 (136)	35.31 ± 0.07 (142)	n.s.	n.s.	n.s.

^a, b^ different letters indicate significant differences (*p* < 0.05) between compartments within the same feeding group. * indicate significant differences (*p* < 0.05) between feeding groups within the same compartment.

**Table 6:** Body condition values and body surface temperatures (mean ± SE (*n*)) for compartment II and III on measurement day 3, according to the feeding groups.

	Comp. II		Comp. III		*p*-Values (ANOVA)		
	Heavy/Lean	Heavy/Fat	Light/Lean	Light/Fat	Comp.	Feeding Group	Comp. × Feeding Group
Backfat (cm)	1.33 ± 0.03 (76)	1.42 ± 0.03 (69) *	1.28 ± 0.02 (107)	1.37 ± 0.03 (106) *	0.07	0.002	n.s.
Muscle (cm)	5.05 ± 0.07 (52)	4.88 ± 0.08 (41) *	5.12 ± 0.05 (75)	4.93 ± 0.06 (71) *	n.s.	0.01	n.s.
Backfat/Muscle	0.252 ± 0.007 (52)	0.288 ± 0.007 (41) *	0.241 ± 0.005 (75)	0.274 ± 0.008 (71) *	0.09	0.001	n.s.
Bodyweight (kg)	113.6 ± 0.7 (78) ^a^	113.7 ± 0.9 (70) ^a^	106.2 ± 0.7 (110) ^b^	107.1 ± 0.8 (107) ^b^	0.001	n.s.	n.s.
Reference Temp. (°C)	33.63 ± 0.23 (65)	32.98 ± 0.26 (58)	32.86 ± 0.21 (86)	33.22 ± 0.24 (77)	n.s.	n.s.	0.06
Min. Temp. ME (°C)	23.16 ± 0.19 (57)	22.60 ± 0.25 (51)	22.73 ± 0.23 (74)	22.97 ± 0.23 (63)	n.s.	n.s.	0.08
Mean. Temp. ME (°C)	30.51 ± 0.19 (57)	30.18 ± 0.20 (51)	30.17 ± 0.19 (74)	30.46 ± 0.19 (63)	n.s.	n.s.	n.s.
Max. Temp. ME (°C)	33.13 ± 0.13 (57)	32.79 ± 0.16 (51)	32.95 ± 0.13 (74)	33.18 ± 0.14 (63)	n.s.	n.s.	0.05
Min. Temp. SC (°C)	29.70 ± 0.27 (57)	29.31 ± 0.26 (51)	29.15 ± 0.22 (75)	29.53 ± 0.25 (63)	n.s.	n.s.	n.s.
Mean Temp. SC (°C)	31.31 ± 0.19 (57)	31.10 ± 0.24 (51)	30.99 ± 0.17 (75)	31.03 ± 0.25 (63)	n.s.	n.s.	n.s.
Max. Temp. SC (°C)	32.37 ± 0.16 (57)	32.24 ± 0.23 (51)	32.14 ± 0.16 (75)	32.28 ± 0.17 (63)	n.s.	n.s.	n.s.

^a, b^ different letters indicate significant differences (*p* < 0.05) between compartments within the same feeding group. * indicate significant differences (*p* < 0.05) between feeding groups within the same compartment.

## Supplementary Materials

The following are available online at https://www.mdpi.com/1424-8220/20/18/5221/s1, Table S1: Chemical composition of supplementary feed “SF1”, and “SF2”, as well as “Soybean Oil”, according to the declaration (88% dry matter content; DM). Triticale whole-plant-silage (WPS) and the Corn-Cob-Mix (CCM) were analyzed in the Institute for Animal Nutrition Hanover (88% DM).

## References

[1] Umweltbundesamt Ammoniak-Emissionen. https://www.umweltbundesamt.de/daten/luft/luftschadstoff-emissionen-in-deutschland/ammoniak-emissionen.

[2] Statistisches Bundesamt 41311-0003. https://www-genesis.destatis.de/genesis//online/data?operation=table&code=41311-0003&levelindex=0&levelid=1571559630955.

[3] Verordnung über Die Anwendung von Düngemitteln, Bodenhilfsstoffen, Kultursubstraten und Pflanzenhilfsmitteln Nach Den Grundsätzen der Guten Fachlichen Praxis Beim Düngen (Düngeverordnung—DüV) in der Fassung der Bekanntmachung Vom 26. Mai 2017 (BGBI. I S. 1305), Die Durch Artikel 1 der Verordnung Vom 28 April 2020 (BGBI. I S. 846) Geändert Worden Ist. https://www.gesetze-im-internet.de/d_v_2017/BJNR130510017.html.

[4] Aarnink A.J.A. (1997). Ammonia Emissions from Houses for Growing Pigs as Affected by Pen Design, Indoor Climate and Behaviour. Ph.D. Thesis.

[5] Kuratorium für Technik und Bauwesen in der Landwirtschaft e.V. (2011). Mastschweinehaltung Mit Sortierschleuse. Verfahren-Kosten-Bewertung.

[6] Reckels B., Hölscher R., Schwennen C., Lengling A., Stegemann U., Waldmann K.-H., Visscher C. (2020). Resource-Efficient Classification and Early Predictions of Carcass Composition in Fattening Pigs by Means of Ultrasound Examinations. Agriculture.

[7] Lengling A., Reckels B., Schwennen C., Hölscher R., Waldmann K.-H., Visscher C., Büscher W. (2020). Validation of a New Resource-Efficient Feeding System for Fattening Pigs Using Increased Crude Fiber Concentrations in Diets: Feed Intake and Ammonia Emissions. Animals.

[8] Cielejewski H., Tholen E., Geerdes K., Leuschner P. Untersuchungen über die Eignung des Videobildsystems opti-SORT (Firma Hölscher + Leuschner) zur Bestimmung des Gewichts und der Beurteilung der AutoFOM-Schlachtkörperqualität von Schweinen in der Endmast. Proceedings of the 7th Tagung Bau, Technik und Umwelt in der Landwirtschaftlichen Nutztierhaltung 2005.

[9] Ebertz P. (2020). Untersuchungen zur Interaktion von Tierwohl und Umweltschutz am Beispiel der Gruppenhaltung von Wartesauen. Ph.D. Thesis.

[10] Shekhawat R.S. (2016). Infrared Thermography—A Review. Int. J. Eng. Trends Technol..

[11] Traulsen I., Naunin K., Müller K., Krieter J. (2010). Untersuchung zum Einsatz der Infrarotthermographie zur Messung der Körpertemperatur bei Sauen. Züchtungskunde.

[12] Loughmiller J.A., Spire M.F., Tokach M.D., Dritz S.S., Nelssen J.L., Goodband R.D., Hogge S.B. (2005). An Evaluation of Differences in Mean Body Surface Temperature with Infrared Thermography in Growing Pigs Fed Different Dietary Energy Intake and Concentration. J. Appl. Anim. Res..

[13] Loughmiller J.A., Spire M.F., Dritz S.S., Fenwick B.W., Hosni M.H., Hogge S.B. (2001). Relationship between mean body surface temperature measured by use of infrared thermography and ambient temperature in clinically normal pigs and pigs inoculated with Actinobacillus pleuropneumoniae. Am. J. Vet. Res..

[14] Gerß H. (2014). Anwendung der Infrarotthermographie zur Nicht-Invasiven Detektion Fieberhafter Tiere in Schweinegruppen—Einschätzung der Anwendbarkeit im Tierseuchenkrisenfall am Beispiel der Klassischen Schweinepest. Ph.D. Thesis.

[15] Cohen M.L. (1977). Measurement of the thermal properties of human skin. A Review. J. Investig. Dermatol..

[16] Lipkin M., Hardy J.D. (1954). Measurement of Some Thermal Properties of Human Tissues. J. Appl. Physiol..

[17] Henriques F.C., Moritz A.R. (1947). Studies of thermal injury. I. The conduction of heat to and through skin and the temperatures attained therein: A theoretical and an experimental investigation. Am. J. Pathol..

[18] Breuer H. (1924). The thermal conductivity of muscle and fat. Pflüg. Arch. Ges. Physiol..

[19] Giering K., Minet O., Lamprecht I., Müller G. (1995). Review of thermal properties of biological tissues. Proc. SPIE.

[20] Schmidt M., Lahrmann K.-H., Ammon C., Berg W., Schön P., Hoffmann G. (2013). Assessment of body temperature in sows by two infrared thermography methods at various body surface locations. J. Swine Health Prod..

[21] Verordnung Zum Schutz Landwirtschaftlicher Nutztiere und Anderer Zur Erzeugung Tierischer Produkte Gehaltener Tiere Bei Ihrer Haltung (Tierschutz-Nutztierhaltungsverordnung—TierSchNutztV) in der Fassung der Bekanntmachung Vom 22 August 2006 (BGBI. I S. 2043), die Zuletzt Durch Artikel 3 Absatz 2 Des Gesetzes Vom 30 June 2017 (BGBI. I S. 2147) Geändert Worden Ist. https://www.gesetze-im-internet.de/tierschnutztv/BJNR275800001.html.

[22] Röhlinger P., Günther M., Danz J., Lyhs L., Zimmerhackel M. (1979). Zur Anwendung der Infrarottechnik in der Veterinärmedizin. (Übersichtsbeitrag). Arch. Exp. Vet..

[23] Clark J.A., Cena K. (1977). The potential of infra-red thermography in veterinary diagnosis. Vet. Rec..

[24] Meola C., Meola C. (2012). Infrared Thermography Recent Advances and Future Trends.

[25] Schaefer A.L., Cook N., Tessaro S.V., Deregt D., Desroches G., Dubeski P.L., Tong A.K.W., Godson D.L. (2004). Early detection and prediction of infection using infrared thermography. Can. J. Anim. Sci..

[26] Glas A. (2008). Vergleichende Untersuchung Klinisch Gesunder und Mit Escherichia coli Infizierter Euterviertel von Kühen Mittels Infrarotthermographie. Ph.D. Thesis.

[27] Menzel A. (2014). Die Eignung von Infrarotthermographie zur Diagnostik von Lungenerkrankungen bei Schweinen. Ph.D. Thesis.

[28] Bianca W. (1976). The significance of meteorology in animal production. Int. J. Biometeorol..

[29] Spiegel F. (2016). Vergleichende Infrarotthermographische und Bakteriologische Untersuchungen am Gesunden Sowie Durch Mastitis Veränderten Gesäuge Beim Schwein. Ph.D. Thesis.

[30] Zhang Z., Zhang H., Liu T. (2019). Study on body temperature detection of pig based on infrared technology: A review. Artif. Intell. Agric..

[31] Schnurrbusch U. (2004). Bedeutung des Körperfettes für die Fruchtbarkeit von Sauen. Lohmann Inf..

[32] Jiang L.J., Ng E.Y.K., Yeo A.C.B., Wu S., Pan F., Yau W.Y., Chen J.H., Yang Y. (2005). A perspective on medical infrared imaging. J. Med. Eng. Technol..

[33] Berry R.J., Kennedy A.D., Scott S.L., Kyle B.L., Schaefer A.L. (2003). Daily variation in the udder surface temperature of dairy cows measured by infrared thermography: Potential for mastitis detection. Can. J. Anim. Sci..

