# Supplementary material for: Feasibility Study on the Use of Infrared Thermography to Classify Fattening Pigs into Feeding Groups According Their Body Composition
Source: Sensors (Basel). 2020 Sep 13;20(18):5221. doi: 10.3390/s20185221 (PMC7571044; doi:10.3390/s20185221)
Supplement: Supplementary file 1 [file sensors-20-05221-s001.pdf]

Table S1. Chemical composition of supplementary feed "SF1", and "SF2", as well as "Soybean Oil", according to the declaration (88% dry matter content; DM). Triticale whole-plant-silage (WPS) and the Corn-Cob-Mix (CCM) were analyzed in the Institute for Animal Nutrition Hanover (88% DM).

Ingredients	SF1 (%)	SF2 (%)	Soybean Oil (%)	Triticale-WPS (%)	CCM (%)
Crude protein	25.00	21.50	0.00	7.10	9.12
Crude fat	3.50	3.00	97.00	1.76	4.11
Crude fiber	7.00	7.00	0.00	20.50	1.28
Crude ash	9.00	7.50	0.00	4.51	1.35
Starch	22.52	21.47	0.00	23.44	60.54
Sugar	6.04	5.17	0.00	1.33	11.00
Lysine	2.20	1.40	0.00	0.21	0.25
Methionine	0.35	0.40	0.00	0.07	0.20
Calcium	1.60	1.00	0.00	0.27	0.06
Phosphorus	0.60	0.65	0.00	0.20	0.31
Acid detergent fibre (ADF)	9.24	10.32	0.00	27.02	2.88
Metabolic energy (megajoule kg^{-1})	11.90	11.60	35.88	6.09	16.00
